# Natural selection on plant resistance to herbivores in the native and introduced range

**DOI:** 10.1093/aobpla/plv090

**Published:** 2015-07-23

**Authors:** Pedro L. Valverde, Juan Arroyo, Juan Núñez-Farfán, Guillermo Castillo, Adriana Calahorra, Rocío Pérez-Barrales, Rosalinda Tapia-López

**Affiliations:** 1 Departamento de Biología, Universidad Autónoma Metropolitana-Iztapalapa, Apartado Postal 55-535, Mexico 09340, Distrito Federal, Mexico; 2 Departamento de Biología Vegetal y Ecología, Universidad de Sevilla, Apartado 1095, Sevilla 41080, Spain; 3 Laboratorio de Genética Ecológica y Evolución, Departamento de Ecología Evolutiva, Instituto de Ecología UNAM, México 04510, Distrito Federal, México

**Keywords:** *Datura stramonium*, enemy release hypothesis (ERH), invasive species, natural selection, plant defence, resistance to herbivores, Spain, specialist and generalist herbivores

## Abstract

When plants are introduced into new regions, the absence of their co-evolved natural enemies can result in lower levels of attack. As a consequence of this reduction in enemy pressure, plant performance may increase and selection for resistance to enemies may decrease. In the present study, we compared leaf damage, plant size and leaf trichome density, as well as the direction and magnitude of selection on resistance and plant size between non-native (Spain) and native (Mexico) populations of *Datura stramonium*. This species was introduced to Spain about five centuries ago and constitutes an ideal system to test four predictions of the enemy release hypothesis. Compared with native populations, we expected Spanish populations of *D. stramonium* to have (i) lower levels of foliar damage; (ii) larger plant size; (iii) lower leaf trichome density that is unrelated to foliar damage by herbivores; and (iv) weak or no selection on resistance to herbivores but strong selection on plant size. Our results showed that, on average, plants from non-native populations were significantly less damaged by herbivores, were less pubescent and were larger than those from native populations. We also detected different selection regimes on resistance and plant size between the non-native and native ranges. Positive selection on plant size was detected in both ranges (though it was higher in the non-native area), but consistent positive selection on relative resistance was detected only in the native range. Overall, we suggest that changes in selection pressure on resistance and plant size in *D. stramonium* in Spain are a consequence of ‘release from natural enemies’.

## Abstract (precis)

Plants introduced into a new range are expected to harbour fewer specialized herbivores and to receive less damage than conspecifics in native ranges. *Datura stramonium* was introduced in Spain about five centuries ago. Here, we compare damage by herbivores, plant size, and leaf trichomes between plants from non-native and native ranges and perform selection analyses. Non-native plants experienced much less damage, were larger and less pubescent than plants of native populations. While plant size was related to fitness in both ranges, selection to increase resistance was only detected in the native region. We suggest this is a consequence of a release from enemies in this new environment.

## Introduction

The occurrence of biological invasions has increased dramatically over the past few decades (Vitousek *et al*. 1996; Sakai *et al*. 2001). This increase has been attributed to human activities (i.e. global trade and transport) extending the range of distribution of many species to novel areas (Bossdorf *et al*. 2005; Genton *et al*. 2005; Hierro *et al*. 2005). Besides their economic impact (Drake *et al*. 1989; Pimentel *et al*. 2000; Sakai *et al*. 2001), biological invasions are recognized as one of the greatest threats to biodiversity and the integrity of natural ecosystems (Sala *et al*. 2000; Sakai *et al*. 2001; Wolfe *et al*. 2004; Agrawal *et al*. 2005; Thelen *et al*. 2005). Nonetheless, not all alien introductions become successful invasions (Groves 1986, 1991; van Kleunen and Johnson 2007; Moles *et al*. 2008). When invasive species are transported to new areas, their abundance and performance will be affected by a different set of parameters than in their native range (Maron *et al*. 2004; Hierro *et al*. 2005). Thus, a relevant question is to determine the conditions that allow alien species become successfully established. Many studies have shown that most successful invasions occur in disturbed habitats (Sax and Brown 2000; Moles *et al*. 2008) and/or where environmental conditions such as the availability of resources (Moles *et al*. 2008) have been altered by human activities. However, only a small fraction of introduced plant species become invasive (Joshi and Vrieling 2005). To invade, alien plants must be able to establish and successfully compete with resident species or occupy empty niches, which may depend on, among other factors, release from their natural enemies (i.e. herbivores, pathogens and parasites) in the non-native habitat (Sax and Brown 2000; Colautti *et al*. 2004; Wolfe *et al*. 2004; Moles *et al*. 2008).

The enemy release hypothesis (ERH, Elton 1958; Crawley 1987; Keane and Crawley 2002) is one of the most commonly considered explanations for the success of invasive plant species (reviewed by Colautti *et al*. 2004; Liu and Stiling 2006). The ERH postulates that during the introduction into a novel region, plant populations experience a decrease in regulation by their co-evolved natural enemies (Keane and Crawley 2002). This liberation from natural enemies can result in reduced levels of herbivory and parasitism in introduced compared with native plant species (Keane and Crawley 2002; Agrawal *et al*. 2005; Mitchell *et al*. 2006), which should result in increased plant size, fecundity (Crawley 1987; Blossey and Notzold 1995; Keane and Crawley 2002; Jakobs *et al*. 2004; Joshi and Vrieling 2005) and population growth in an invasive species’ introduced range compared with its native range (Crawley 1987; Keane and Crawley 2002; Maron *et al*. 2004; Vilà *et al*. 2005; Dawson *et al*. 2014). However, this plastic response to the release from damage by herbivores may also have evolutionary consequences (Jakobs *et al*. 2004; Maron *et al*. 2004; Wolfe *et al*. 2004). If specialist herbivores of an alien plant species are absent in the introduced areas (Keane and Crawley 2002), it is expected that selection for resistance against them may also be absent (van Kleunen and Schmid 2003). Although the production of chemical and mechanical defences may be adaptive in the presence of natural herbivores, the expression of plant resistance to herbivores can be costly when they are scarce or absent (Purrington 2000; Koricheva 2002; Strauss *et al*. 2002; Wolfe *et al*. 2004). For this reason, natural selection in the new environment would reduce resource allocation to defence against herbivores and favour genotypes with improved competitive abilities (i.e. increasing vegetative growth or reproductive effort) (Blossey and Notzold 1995). This is the essence of the ‘Evolution of Increased Competitive Ability (EICA)’ hypothesis proposed by Blossey and Notzold (1995). Obviously, ERH and EICA are not mutually exclusive hypotheses and can be seen as a two-step process, where increased ecological performance follows rapid evolutionary change.

*Datura stramonium* (Solanaceae) is an annual weed that inhabits open, cultivated and disturbed sites where it grows to an average height of 1 m (Núñez-Farfán 1991). *Datura stramonium* is native to Mexico (Jiao *et al*. 2002) and it is widely distributed in warm regions around the world (van Kleunen *et al*. 2007). In its native range, this species is attacked by a wide variety of herbivores (Núñez-Farfán 1991) and leaf damage significantly reduces plant fitness (Núñez-Farfán and Dirzo 1994; Fornoni *et al*. 2003; Valverde *et al*. 2003). Previous field and experimental studies have found that leaf trichomes and tropane alkaloids are two defensive traits that prevent herbivory (Shonle and Bergelson 2000; Valverde *et al*. 2001; Castillo *et al*. 2013; Kariñho-Betancourt and Núñez-Farfán 2015). Moreover, significant selection on resistance (measured as 1—damage) and on defensive traits (trichomes and alkaloids) had been detected in natural and experimental populations (Núñez-Farfán and Dirzo 1994; Núñez-Farfán *et al*. 1996; Shonle and Bergelson 2000; Valverde *et al*. 2001, 2003; Fornoni *et al*. 2004; Castillo *et al*. 2014). In the present study, we investigated the case of *D. stramonium* in Spain, a country where it was introduced about five centuries ago and is presently considered an invasive species (Dana-Sánchez *et al*. 2004; Sanz-Elorza *et al*. 2004). Owing to early trade across the Atlantic, this introduction and invasion were probably the first intercontinental invasion achieved by the species, which actually faced a new biotic environment. If this is the case, Spanish populations have had the highest number of generations in their new range, and therefore the most opportunity for evolutionary change. *Datura stramonium* is therefore an ideal system in which to test some of the predictions of the ERH. In order to accomplish this, we conducted a field survey in southern Spain to examine leaf damage, plant size and trichome density and compare these characters with Mexican populations. We also compared phenotypic selection gradients on resistance and plant size in this species’ non-native and native ranges. In this study, we tested four predictions of the ERH. If specialist herbivore insects are absent in the new region (Keane and Crawley 2002), we predicted that populations in Spain would have (i) lower levels of foliar damage and (ii) larger plant size. We also expected that since resistance traits can be costly (Strauss *et al*. 2002), trichome density would be lower and unrelated to foliar damage by herbivores in Spain. Finally, we expected weak or no selection on resistance to herbivores in the non-native region but strong selection on plant size. Recently, several studies evaluating invasive plant species in their native and introduced ranges with natural populations have found evidence that supports some ecological expectations of the ERH (e.g. Memmott *et al*. 2000; Wolfe 2002; Jakobs *et al*. 2004; Blaisdell and Roy 2014; Huberty *et al*. 2014; Kambo and Kotanen 2014). However, to our knowledge, this is one of the first attempts to evaluate the joint pattern of selection on plant resistance to herbivores and plant size in the non-native and native range.

## Methods

### Study species

*Datura stramonium* (Solanaceae) is a cosmopolitan annual weed occurring in a wide variety of plant communities in North America (Avery *et al*. 1959; Weaver and Warwick 1984), and Mexico is likely to be its centre of origin (Symon and Haegi 1991; Jiao *et al*. 2002). In its native range, this herbaceous plant inhabits open, cultivated lands, roadsides and disturbed sites (Núñez-Farfán 1991). In central Mexico, leaves of this species are consumed by at least two specialist herbivorous beetles, *Epitrix parvula* and *Lema daturaphila* (Coleoptera: Chrysomelidae), and by a generalist grasshopper, *Sphenarium purpurascens* (Orthoptera: Pyrgomorphidae) (Núñez-Farfán and Dirzo 1994). *Datura stramonium* is also attacked by a specialist pre-dispersal seed predator, *Trichobaris soror* (Coleoptera: Curculionidae) (Cabrales-Vargas 1991; Bello-Bedoy *et al*. 2011). A complete description of the plant, herbivorous insects and damage types produced by them can be found elsewhere (Núñez-Farfán 1991; Núñez-Farfán and Dirzo 1994; Carmona and Fornoni 2013). Previous studies in natural and experimental populations of *D. stramonium* in their native range have reported that foliar damage caused by herbivorous insects imposes selection on resistance and/or tolerance (Núñez-Farfán and Dirzo 1994; Shonle and Bergelson 2000; Valverde *et al*. 2001, 2003; Fornoni *et al*. 2004). Moreover, leaf trichomes and tropane alkaloids, two putative components of defence, can evolve as a result of natural selection imposed by herbivorous insects (Shonle and Bergelson 2000; Castillo *et al*. 2014; Kariñho-Betancourt and Núñez-Farfán 2015).

*Datura stramonium* is an invasive species in almost all temperate and tropical regions of the world (van Kleunen *et al*. 2007). In Spain, *D. stramonium* was introduced, probably from Mexico, by Post-Columbian expeditionists between the years 1540 and 1577 (Sanz-Elorza *et al*. 2004). In this country *D. stramonium* is considered an invasive species since it mainly inhabits agricultural fields, waste lands and natural habitats like riparian areas and wetlands in warm regions with moderate to high human influence. Currently, the main problem is that this species reaches high densities in soils with high nitrogen content that prevent the development of native species (Dana-Sánchez *et al*. 2004; Sanz-Elorza *et al*. 2004).

### Sampling populations and data collection in Spain

From September to November 2010 and 2011, we sampled 14 populations of *D. stramonium* occurring in different habitats and environmental conditions in southern Spain (Table 1). The populations were sampled in the regions of Andalousia, Extremadura and Murcia. While all are described as having a Mediterranean climate, yearly mean rainfall varied by 4-fold among sites, with wettest conditions in the west (more than 800 mm) and driest conditions in the east (∼200 mm, see Table 1). Thus, a wide range of conditions was represented within the widespread Mediterranean climate of the Iberian Peninsula. The linear distance between pairs of populations ranged from 5 to 468 km. In a sample of mature plants in each population (mean sample size: 26.74 ± 1.69 SE individual plants, Table 1), we measured the basal stem diameter as an estimate of plant size, collected a sample of 8–40 fully expanded leaves and collected all the fruits produced.

**Table 1.** Geographic location and environmental characteristics of 14 populations of *D. stramonium* in the non-native range (southern Spain. *n* = sample size). ^1^Data taken from Ninyerola *et al*. (2005). a.s.l, above sea level

Number and locality of each population (Province) (*n*)	Geographical coordinates	Habitat	Altitude (m a.s.l.)	Mean annual precipitation (mm)^1^	Mean annual temperature (°C)^1^
1. Hinojos 1 (Huelva) (18)	37°18′0.39″N 6°22′41.72″W	River bank	67	503.3	18
2. Hinojos 2 (Huelva) (22)	37°19′28.36″N 6°25′32.45″W	River bank	88	515.8	18
3. Bolonia (Cádiz) (30)	36°5′9.99″N 5°46′7.57″W	Stream in seashore	3	693.3	18
4. Gerena (Sevilla) (30)	37°31′28.86″N 6°11′24.76″W	River bank	55	501.6	18
5. Zubia (Granada) (30)	37°7′47.28″N 3°35′57.06″W	Cropland edge	692	337.5	15
6. Castañuelos (Huelva) (9)	37°56′19.83″N 6°35′2.97″W	Oak forest edge	437	825.8	16
7. El Higueral (Almería) (30)	37°23′12.61″N 2°29′56.48″W	Dry riverbed	880	255.0	14
8. Pinilla (Murcia) (30)	37°41′3.10″N 1°17′0.62″W	Wasteland	240	236.6	17
9. Don Fadrique (Granada) (25)	37°57′39.75″N 2°26′8.75″W	Abandoned orchard	1161	422.5	13
10. Lora del Río (Sevilla) (30)	37°39′33.28″N 5°32′5.93″W	River bank	36	483.3	18
11. El Pedroso (Sevilla) (30)	37°50′12.81″N 5°45′58.67″W	Roadside	383	501.6	17
12. Cardeña (Córdoba) (30)	38°14′56.63″N 4°12′58.45″W	Dry riverbed	351	645.8	17
13. Valdeflores (Sevilla) (30)	37°43′2.23″N 6°18′50.44″W	River bank	287	598.2	17
14. Cabeza La Vaca (Badajoz) (30)	38°6′48.00″N 6°24′23.36″W	River bank	548	625.0	16

We measured the total and damaged areas for each collected leaf using free ImageJ v1.47 software (National Institutes of Health, Bethesda, MD, USA). For a given plant *i*, relative resistance to herbivores (*R_i_*) was estimated as the converse of the average relative leaf damage (*D_i_*) as:

$$R_{i}=1-D_{i}=1-\left(\frac{1}{n}\sum_{i=1}^{n}\frac{A_{D}}{A_{T}}\right),$$

where *A*_D_ and *A*_T_ are the damaged and total leaf areas, respectively, and *n* is the number of leaves sampled (following Núñez-Farfán and Dirzo 1994; Bello-Bedoy and Núñez-Farfán 2010). This estimate of resistance to herbivores (*R_i_*) is broadly interpreted as a measure of total resistance (see Leimu and Koricheva 2006) and has been used in previous studies with *D. stramonium* (Núñez-Farfán and Dirzo 1994; Fornoni *et al*. 2003, 2004).

We measured leaf trichome density as the total number of trichomes within a 2.5 mm^2^ area on the basal central area of the adaxial side of the leaf following Valverde *et al*. (2001), using a dissecting microscope. This sampled area of the leaf gives a good estimate of the whole-leaf average trichome density (Valverde *et al*. 2001). In each population, we estimated the average trichome density per plant from a sample of 8–10 fully expanded, mature leaves, obtained from the same sample of leaves used to estimate relative damage.

The number of fruits per plant was used as a measure of individual maternal plant fitness. Since *D. stramonium* has a mixed mating system (Motten and Antonovics 1992; Motten and Stone 2000) with a high level of selfing (Núñez-Farfán *et al*. 1996; van Kleunen *et al*. 2007), the number of fruits and seeds is a good estimator of reproductive success of the female function (see Mauricio and Rausher 1997), and male and female functions are probably highly correlated in selfing plants (Charlesworth and Charlesworth 1981; Bertin 1988).

### Data from Mexican populations

Measurements of leaf damage, plant diameter, leaf trichome density and number of fruits per plant from seven populations of *D. stramonium* in its native range were obtained from a previous study (Castillo *et al*. 2014). These populations occur in different plant communities (Castillo *et al*. 2014). The chosen populations were Acolman, Patria Nueva, San Martín, Sanabria, Santo Domingo, Tzin Tzun Tzan and Valsequillo. The mean sample size was 29 (±1.88 SE) individual plants per population. Data collection procedures in Mexico were similar to those conducted in Spain. Further details on geographic location and environmental characteristics of the seven Mexican populations of *D. stramonium* are available elsewhere (Table S1 in Castillo *et al*. 2014).

### Statistical analysis

We performed a nested analysis of variance to test differences in relative leaf damage, plant diameter and leaf trichome density due to range (non-native vs. native) and population (within range). Range was considered as a fixed factor and population (within range) as a random factor. We used regression analyses to assess the effect of leaf trichome density on relative leaf damage for each population. Prior to analyses, plant diameter and leaf trichome density were natural log-transformed, while relative leaf damage was arcsine-transformed (Sokal and Rohlf 2012).

### Directional selection on relative resistance and plant size

In order to estimate the magnitude and direction of selection gradients (*β_i_*) on relative resistance and plant size, we performed multiple regression analysis (Lande and Arnold 1983) in each population. For these analyses, directional selection gradients (*β_i_*) were estimated as the standardized partial linear regression coefficients of relative fitness as a function of relative resistance and plant diameter. For each population, relative resistance and plant diameter were standardized ($\bar{X}=0$ and *S* = 1) and plant fitness was relativized (the number of fruits divided by the corresponding population mean fitness) (Lande and Arnold 1983).

### Comparing directional selection gradients between ranges

In order to compare the consistency of the patterns of selection on relative resistance and plant size between ranges, we performed a meta-analysis to estimate the effect size of each selection gradient (Castillo *et al*. 2014). The effect size is a value that reflects the strength of a relationship between two variables (Borenstein *et al*. 2009). Mean effect sizes were used to compare estimates of phenotypic selection on relative resistance and/or plant size corresponding to populations of each range. We estimated effect sizes using the partial regression coefficients (i.e. selection gradients) weighted by their variances (following Castillo *et al*. 2014). Assuming that the true effect size varies from population to population, we estimated mean effect sizes and their 95 % confidence intervals by applying a random-effect model (Borenstein *et al*. 2009). If the confidence interval around the mean effect size did not include zero, we concluded that there is a significant effect on the pattern and intensity of selection on relative resistance and/or plant size in a particular range. The meta-analyses were performed using the *metaphor* package (Viechtbauer 2010) for R v3.0.2 software (R Development Core Team 2011).

## Results

### Between-range and among-population variation

**Figure 1.**
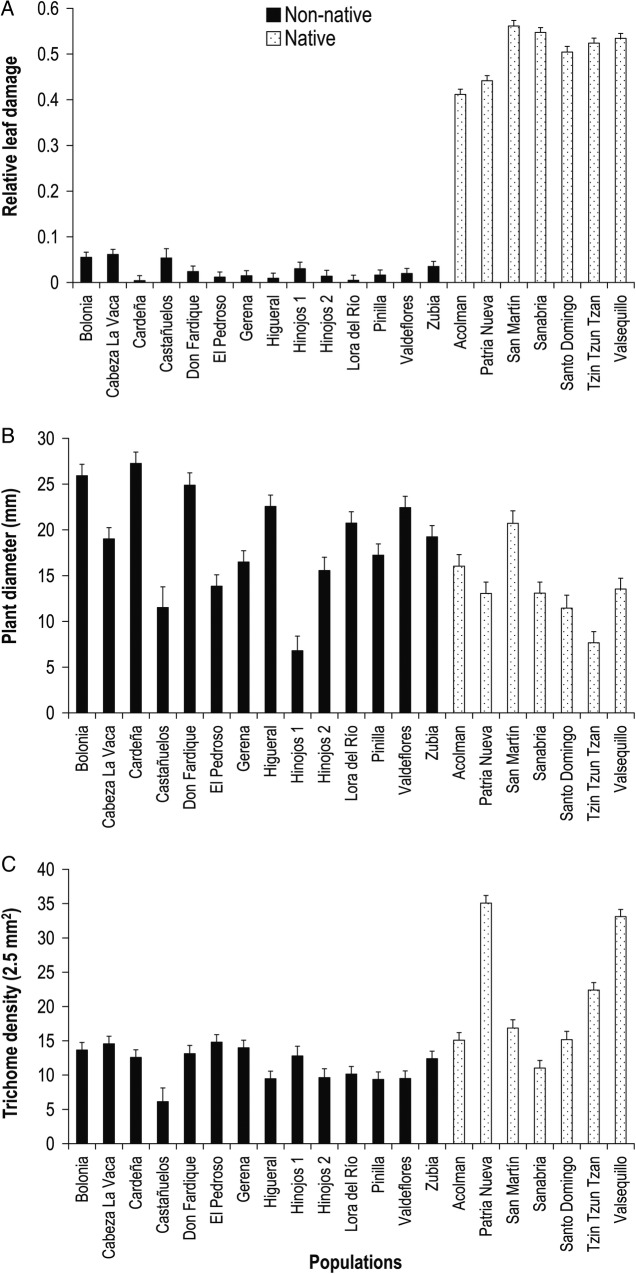
Mean (±SE) relative leaf damage by herbivores (A), plant diameter (B) and leaf trichome density (C) of populations of *D. stramonium* in the non-native (southern Spain) and native (Mexico) ranges.

Relative leaf damage, plant diameter and leaf trichome density differed significantly between the non-native and native ranges and among populations within each range (Fig. 1). In the non-native range, populations experienced low average levels of relative leaf damage ($\bar{X}\pm \text{SE:}$ 0.025 ± 0.0102, Fig. 1A). On average, Spanish populations had 20 times less damage than Mexican populations (0.503 ± 0.013, *F*_1,  19.04_ = 623.07, *P* < 0.0001, Fig. 1A). Similarly, Spanish populations are 1.84 times less pubescent (11.57 ± 1.66 trichomes × 2.5 mm^2^) than Mexican populations (21.24 ± 2.14, *F*_1,  19.03_ = 13.47, *P* = 0.0016, Fig. 1C). Conversely, on average, Spanish plant populations had a significantly larger average diameter than Mexican plant populations (18.81 ± 1.37 and 13.64 ± 1.77 mm, respectively, *F*_1,  19.04_ = 4.54, *P* = 0.0463, Fig. 1B).

In the non-native range, field observations indicated a low richness of phytophagous invertebrates on individual plants of *D. stramonium* (*n* = 6 species, Table 2). The generalist moth *Helicoverpa armigera* (Noctuidae: Lepidoptera) was the main herbivore in all populations. Besides leaves, larvae of *H. armigera* also bored into fruits to consume immature seeds until pupation (P. L. Valverde, pers. obs.).

**Table 2.** Phytophagous invertebrate species sampled on leaves of *D. stramonium* in 14 populations in the non-native range (southern Spain). The number of populations as in Table 1.

Class	Order	Family	Species	Population
Insecta	Lepidoptera	Noctuidae	*Helicoverpa armigera*	1–14
	Hemiptera	Pentatomidae	*Nezara viridula*	11 and 12
		Pyrrhocoridae	*Pyrrhocoris apterus*	11 and 13
	Coleoptera	Curculionidae	*Coniatus repandus*	13
	Orthoptera	Tettigoniidae	*Phaneroptera* sp.	12
Gastropoda	Pulmonata	Helicidae	*Theba pisana*	11

### Effect of leaf trichome density on relative leaf damage

We did not detect a consistent effect of trichome density on relative leaf damage in either range. In the non-native range, one population showed a negative relationship (Castañuelos population, *F*_1,  7_ = 14.13, *P* = 0.0071). In the native range, two populations showed a positive relationship, Patria Nueva (*F*_1,  28_ = 15.19, *P* = 0.0006) and Tzin Tzun Tzan (*F*_1,  29_ = 11.19, *P* = 0.0023), and one showed a negative relationship (San Martín population, *F*_1,  23_ = 10.84, *P* = 0.0032).

### Directional phenotypic selection on relative resistance and plant size

**Figure 2.**
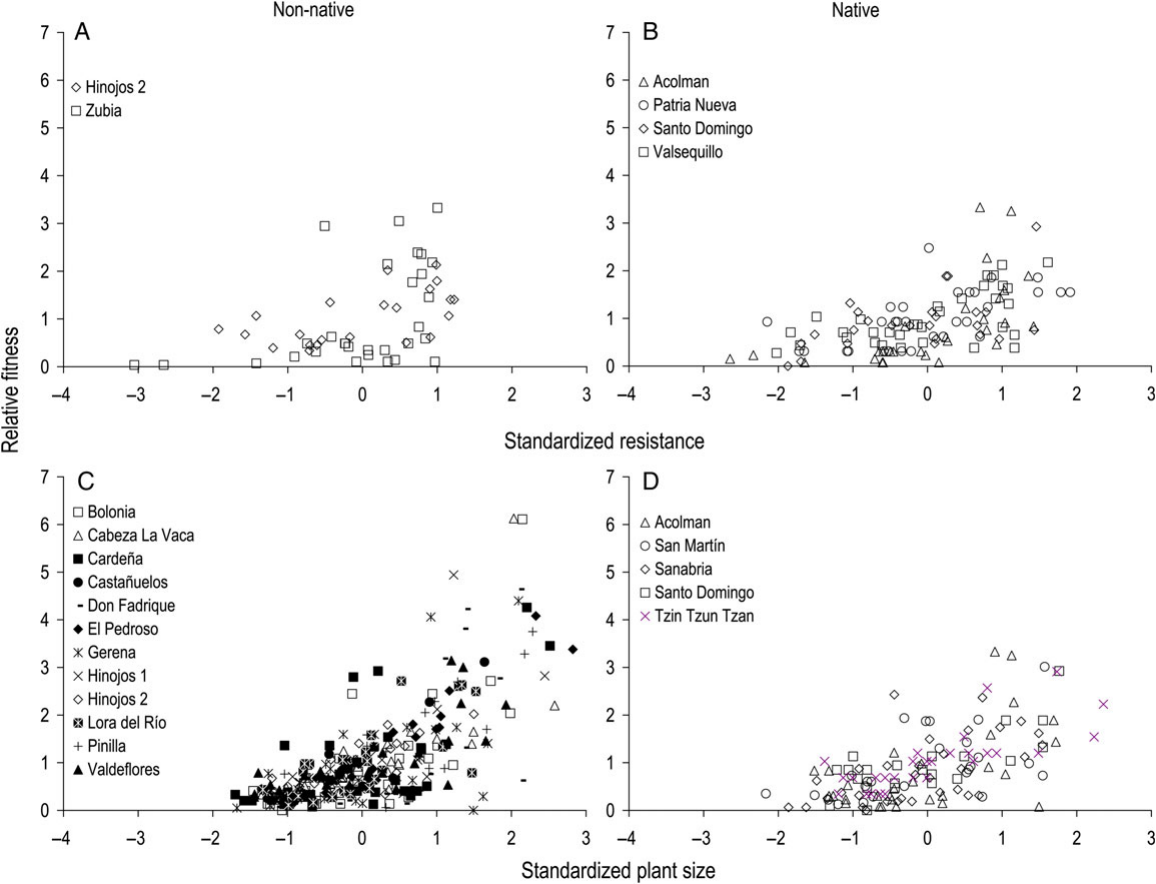
Relationship between standardized relative resistance and relative fitness of populations of *D. stramonium* in the non-native (southern Spain; A) and native (Mexico; B) ranges. Relationship between standardized plant size and relative fitness in the non-native (southern Spain; C) and native (Mexico; D) ranges. Only significant relationships are shown (see Table 3).

We found positive selection on relative resistance in two populations (Table 3, Fig. 2A) and on plant size in 12 populations (Table 3, Fig. 2C) of the 14 populations measured in the introduced range. In the seven native range populations measured, we found positive selection on relative resistance in four populations (Table 3, Fig. 2B) and on plant size in five populations (Table 3, Fig. 2D).

**Table 3.** Standardized directional (*β_i_*) selection gradients (±SE) on relative resistance and plant diameter in populations of *D. stramonium* from the non-native (southern Spain) and native (México) ranges. Significant gradients appear in bold type face.

Range	Population	Plant trait	*β* (±SE)	*t* (df)	*P*
Non-native	Hinojos 1	Relative resistance	0.074 (0.220)	0.34 (15)	0.7422
		Diameter	**0.827 (0.069)**	**3.75 (15)**	**0**.**0019**
	Hinojos 2	Relative resistance	**0.167 (0.075)**	**2.20 (19)**	**0**.**0403**
		Diameter	**0.365 (0.075)**	**4.86 (19)**	**0**.**0001**
	Bolonia	Relative resistance	0.271 (0.164)	1.65 (27)	0.1099
		Diameter	**0.927 (0.164)**	**5.65 (27)**	**<0**.**0001**
	Gerena	Relative resistance	0.026 (0.182)	0.15 (27)	0.8840
		Diameter	**0.551 (0.182)**	**3.02 (27)**	**0**.**0050**
	Zubia	Relative resistance	**0.480 (0.179)**	**2.67 (27)**	**0**.**0126**
		Diameter	−0.013 (0.179)	−0.07 (27)	0.9414
	Castañuelos	Relative resistance	−0.307 (0.243)	−1.26 (6)	0.2533
		Diameter	**0.716 (0.243)**	**2.95 (6)**	**0**.**0257**
	El Higueral	Relative resistance	0.200 (0.207)	0.97 (27)	0.3428
		Diameter	0.191 (0.207)	0.92 (27)	0.3641
	Pinilla	Relative resistance	−0.004 (0.096)	−0.05 (27)	0.9630
		Diameter	**0.810 (0.096)**	**8.37 (27)**	**<0**.**0001**
	Don Fadrique	Relative resistance	0.175 (0.216)	0.81 (22)	0.4261
		Diameter	**0.978 (0.200)**	**4.89 (22)**	**<0**.**0001**
	Lora del Río	Relative resistance	0.036 (0.107)	0.34 (27)	0.7383
		Diameter	**0.545 (0.107)**	**5.07 (27)**	**<0**.**0001**
	El Pedroso	Relative resistance	−0.046 (0.058)	−0.8 (27)	0.4279
		Diameter	**0.954 (0.058)**	**16.43 (27)**	**<0**.**0001**
	Cardeña	Relative resistance	−0.157 (0.156)	−1.01 (27)	0.3217
		Diameter	**0.667 (0.156)**	**4.27 (27)**	**0**.**0002**
	Valdeflores	Relative resistance	0.123 (0.096)	1.27 (27)	0.2135
		Diameter	**0.532 (0.096)**	**5.52 (27)**	**<0**.**0001**
	Cabeza La Vaca	Relative resistance	0.231 (0.153)	1.51 (27)	0.1421
		Diameter	**0.877 (0.153)**	**5.73 (27)**	**<0**.**0001**
Native	Acolman	Relative resistance	**0.336 (0.142)**	**2.58 (26)**	**0**.**0311**
		Diameter	**0.337 (0.147)**	**2.58 (26)**	**0**.**0311**
	Patria Nueva	Relative resistance	**0.343 (0.099)**	**3.45 (27)**	**0**.**0018**
		Diameter	0.042 (0.099)	0.43 (27)	0.6739
	San Martín	Relative resistance	0.145 (0.131)	1.10 (22)	0.2822
		Diameter	**0.376 (0.127)**	**2.94 (22)**	**0**.**0076**
	Sanabria	Relative resistance	0.025 (0.108)	0.24 (29)	0.8157
		Diameter	**0.451 (0.120)**	**3.75 (29)**	**0**.**0008**
	Santo Domingo	Relative resistance	**0.225 (0.091)**	**2.47 (20)**	**0**.**0228**
		Diameter	**0.418 (0.091)**	**4.59 (20)**	**0**.**0002**
	Tzin Tzun Tzan	Relative resistance	0.018 (0.087)	0.21 (28)	0.8325
		Diameter	**0.488 (0.087)**	**5.55 (28)**	**<0**.**0001**
	Valsequillo	Relative resistance	**0.324 (0.081)**	**3.98 (30)**	**0**.**0004**
		Diameter	0.063 (0.081)	0.77 (30)	0.4459

### Comparing directional selection gradients among populations

**Figure 3.**
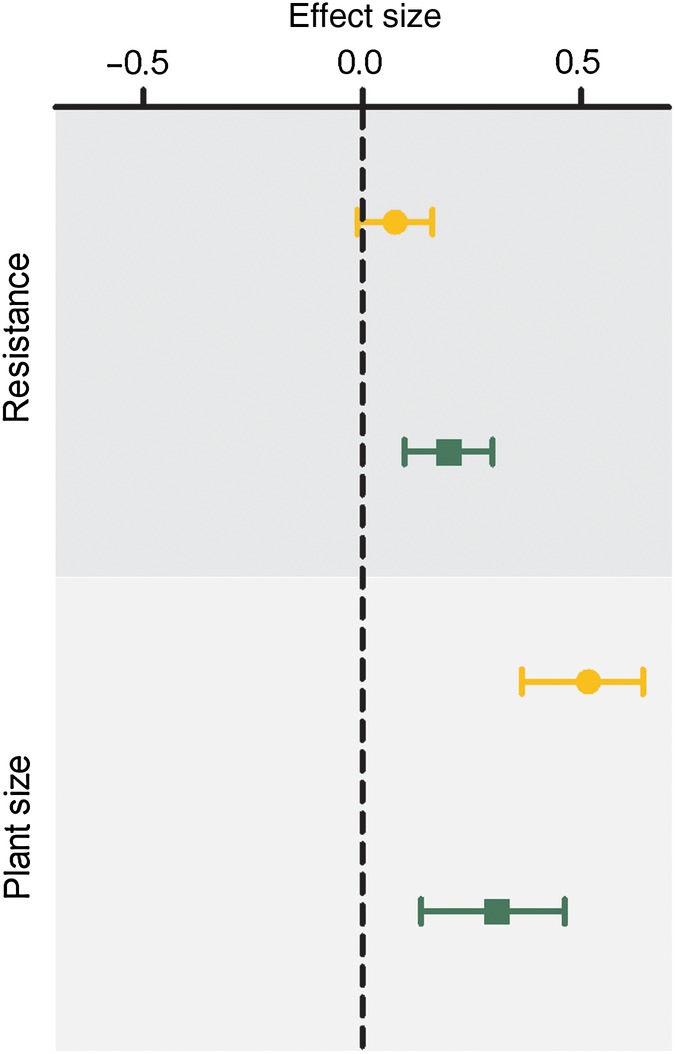
Forest plot of the mean effect sizes and 95 % confidence intervals for standardized selection gradients for relative resistance and plant size (plant diameter). Green squares correspond to the native range and yellow circles to the non-native range.

In the native and non-native ranges, mean effect sizes of selection on plant size were 0.308 (95 % CI 0.135–0.46) and 0.516 (0.365–0.64), respectively (Fig. 3). Given that neither confidence interval overlaps zero, plant size was positively selected in both ranges, though this trend was stronger in the introduced range. On the other hand, mean effect sizes of relative resistance show a different pattern. We detected a consistent trend to positively select relative resistance in the native range (0.199, 95 % CI 0.096–0.297, Fig. 3), whereas no consistent trend was detected in the introduced range (0.075, 95 % CI −0.011–0.16, Fig. 3).

## Discussion

Native and non-native populations of *D. stramonium* experience differential leaf damage by herbivores. In Spain, leaf damage was very low, averaging 2.5 % of total leaf area, thus supporting our first prediction. We also found that plants in the non-native range were larger than those of the native range, supporting our second prediction. In partial support of our third prediction, leaf trichome density was significantly lower in the non-native range. However, although Mexican populations had a higher mean leaf trichome density, its effect on leaf damage was not consistent. Our major finding was the detection of different selection regimes on resistance and plant size between the non-native and native ranges. While a trend of positive selection on plant size was detected in both ranges (though higher in the non-native area), positive selection on relative resistance was only detected in the native region, supporting our fourth prediction. Overall, our study suggests that changes in selection pressures on resistance and plant size in *D. stramonium* in Spain are the consequence of a ‘release from natural enemies’ in this new environment.

Introduced plant species commonly harbour fewer species of specialized natural enemies and are less attacked in their introduced vs. their native ranges (Memmott *et al*. 2000; Wolfe 2002; Torchin and Mitchell 2004; Wolfe *et al*. 2004; Agrawal *et al*. 2005; Genton *et al*. 2005; Blaisdell and Roy 2014). This appears to be the case in *D. stramonium* in southern Spain, where leaf damage was 20 times lower than in Mexico. This difference in leaf damage is in agreement with the ERH. There are at least two non-exclusive factors that could explain the lower leaf damage in the non-native area. First, the three specialist herbivores of *D. stramonium* commonly found in the native range are absent in populations of southern Spain. This is consistent with one of the basic predictions of ERH and is one of the main explanations cited for the success of introduced invasive plants species (Liu and Stiling 2006). Second, the generalist herbivores recorded during our field surveys in Spain (cf. Table 2; Torres-Vila *et al*. 2000; Torres-Vila *et al*. 2002; Ribes *et al*. 2004; Mata *et al*. 2013) consume a very small amount of foliar tissue of *D. stramonium*, and are therefore unlikely to exert strong selective pressure for plant resistance in the new environment.

Leaf trichome density is a plant resistance trait that prevents damage by herbivores (Levin 1973; Johnson 1975; Rodriguez *et al*. 1984; Handley *et al*. 2005; Holeski 2007), and previous studies on *D. stramonium* in its native range support this defensive function (Valverde *et al*. 2001; Kariñho-Betancourt and Núñez-Farfán 2015). Populations of *D. stramonium* surveyed in Spain were significantly less pubescent than populations in the native range. However, we did not detect a consistent effect of leaf trichomes on leaf damage by herbivores in the native or non-native areas. In this sense, the variation in leaf trichome density may be neutral in relation to resistance or related to the variation in other environmental factors (Valverde *et al*. 2001; Kariñho-Betancourt and Núñez-Farfán 2015). For instance, leaf trichomes might reduce water loss in dry environments (Turner and Kramer 1980; Fitter and Hay 1987; Larcher 2001). Further studies are therefore needed to explore whether the reduction in leaf trichomes or other components of defence in the non-native range is actually a consequence of relaxed selection pressure for resistance to generalist herbivores.

The ERH posits that plants introduced into a new range benefit from the absence of their natural enemies, resulting in larger and more vigorous plants than in their native ranges (Crawley 1987; Maron and Vilà 2001; Keane and Crawley 2002; Jakobs *et al*. 2004). Reduction of damage by herbivores would translate into higher allocation to plant growth in the non-native range (Keane and Crawley 2002; Vilà *et al*. 2005). Consistent with this idea, our study revealed that plants of *D. stramonium* in the non-native area are larger than in the native region. Furthermore, a reduction of resource allocation to defence in the new environment (where specialist herbivores are absent) would select for allocation to traits that enhance plants' competitive ability (Blossey and Notzold 1995). Our analysis of selection gradients on plant size showed that this trait was consistently favoured among populations of *D. stramonium* in both ranges. However, the mean effect size of selection gradients on plant size was higher in the non-native range, suggesting stronger selection. In this sense, it has been suggested that plant size promotes competitive ability (Kelly 1992; Cahill *et al*. 2008). In addition, our study also supports the prediction that plants from the introduced area experience weak or no selection on resistance to herbivores since the mean effect size of selection gradients on resistance revealed a no consistent trend in the non-native range. In contrast, mean effect size for selection to resistance showed a consistent positive trend in the native range.

## Conclusions

Since *D. stramonium* has experienced a much larger number of generations than minimum estimated for evolutionary change in a new range (Prentis *et al*. 2008), this constitutes an opportunity for selection to occur. Our study suggests that *D. stramonium* is not subject to demographic regulation by generalist herbivores in the new environment and that selection would not favour the maintenance of allocation to defence in Spain. Hence, a variation in resistance to herbivores among populations of *D. stramonium* in the new geographic range may not be adaptive. On the other hand, if genetic differentiation for traits that enhance competitive ability in the introduced range occur, and these are beneficial under the novel selective scenario (Bossdorf *et al*. 2005), further studies warrant genetic variation and selection. Such evidence will help us to better understand the adaptive evolutionary change in the introduced populations of *D. stramonium* in Spain.

## Sources of Funding

This research was funded by the Universidad Autónoma Metropolitana-Iztapalapa, the Universidad de Sevilla and the PAPIIT UNAM grant IN212214.

## Contributions by the Authors

P.L.V., J.A. and J.N-F. designed the study. P.L.V., J.A. and R.P-B. carried out the field work. P.L.V., J.N-F., A.C., G.C. and R.T-L. performed the statistical analyses. P.L.V., J.A. and J.N-F. drafted the manuscript. All authors read and approved the manuscript.

## Conflict of Interest Statement

None declared.
